# Infantile Hemangioma Treated with Propranolol Readmission Trends, Complications of Therapy, and Cost: A PHIS Database Study

**DOI:** 10.1155/2022/4423558

**Published:** 2022-09-09

**Authors:** Muhammad Abu-Rmaileh, Hayden C. Hairston, Isabella Zaniletti, Anvesh Kompelli, Kyle P. Davis, James Reed Gardner, Elijah H. Bolin, Gresham T. Richter

**Affiliations:** ^1^College of Medicine, University of Arkansas for Medical Sciences, Little Rock, AR, USA; ^2^Children's Hospital Association, Biostatistician, Lenexa, KS, USA; ^3^Department of Otolaryngology-Head and Neck Surgery, University of Arkansas for Medical Sciences, Little Rock, AR, USA; ^4^Department of Pediatrics, Section of Pediatric Cardiology, University of Arkansas for Medical Sciences, USA

## Abstract

**Objective:**

To examine admission trends, complications, and costs for inpatient infantile hemangioma (IH) associated with propranolol therapy utilizing the Pediatric Health Information System (PHIS) database. *Study Design*. A retrospective cohort study was completed using the PHIS database. The PHIS database was queried from 2008 to 2020 for children without cardiac disease and between the ages of three weeks and one year who were admitted with a diagnosis of IH and administered propranolol. Admissions were trended annually and by geographic region. Primary outcomes were length of stay (LOS), readmission, mortality, propranolol-related complications, and costs. Bivariate and multivariable analyses were employed to identify predictors of the primary outcomes.

**Results:**

A total of 2290 unique patient encounters were identified. Admissions steadily decreased after 2011, with variations by geographic region. There was no mortality and only 60 (2.6%) propranolol-related complications. African-American race (odds ratio (OR) 1.20 [95% CI: 1.02-1.41]), respiratory comorbidities (OR 2.04 [95% CI: 1.42-2.93]), neurologic conditions (OR 1.34 [95% CI: 1.09-1.59]), admission to an intensive care unit (OR 1.31 [95% CI: 1.09-1.59]), bronchospasm (OR 1.37 [95% CI: 1.22-1.55]), and hyperkalemia (OR 1.86 [95% CI: 1.08-3.20]) were associated with increased LOS. Neurologic conditions (OR 2.87 [95% CI: 1.76-4.67]) and respiratory comorbidities (OR 2.48 [CI: 1.43-4.30]) were associated with readmission. Average cost per admission was $5,158 ($3,259 to $8,560 range).

**Conclusion:**

There is an overall national decline in rate of admissions for IH propranolol therapy. Inpatient admission may be beneficial for patients with neurologic or respiratory conditions.

## 1. Introduction

Infantile hemangiomas (IH) are the most common benign vascular tumor in children. The prevalence has ranges between 4–5% of newborns and 20–30% of premature infants [1–3]. Their development involves a proliferative growth phase lasting up to 12 months of age followed by slow involution. In 40% of children, IHs resolve spontaneously without complication. However, approximately 12–24% of children with IH have complications requiring treatment; these complications include ulceration, bleeding, deformity, functional compromise, and fibrofatty residua [2, 4]. By treating early, clinicians can prevent avoidable sequela of a “problematic” IH. Historically, surgical excision, laser therapy, and intralesional injections have been the mainstay of treatment.

In 2008, propranolol, a lipophilic nonselective *β*-blocker, was discovered to halt IH growth and lead to early involution. Since then, numerous reports have demonstrated its efficacy, potential mechanisms, and risks in treating infants with IH. Currently, propranolol has become first-line treatment for “problematic” IHs [5]. Longitudinal evidence suggests that this is relatively safe with a limited side effect profile but with some risks in select populations [6]. The high liposolubility of propranolol leads to passage through the blood-brain barrier that may cause central nervous system side effects, such as agitation and sleep disturbances [6]. Other side effects such as bronchospasm or bronchial hyperreactivity and hypoglycemia are direct effects of *β*_2_-adrenergic receptor (*β*_2_-AR) blockade caused by propranolol [5]. Intolerable side effects have been reported leading to therapeutic cessation and a higher incidence of rebound tumor growth [6, 7].

There is limited understanding of how comorbid conditions affect admission mortality, readmission, and length of stay (LOS) in infants with problematic IH treated with propranolol. Knowing possible impedances to discharge and mortality can improve outcomes while practicing cost-effective medicine. In a multi-institution cohort using the Pediatric Health Information System® (PHIS), we sought to determine national and regional historical trends and complications from inpatient admission for IH with associated propranolol treatment.

## 2. Methods

### 2.1. Study Design and Data Source

We performed a retrospective cohort study using the PHIS (Children's Hospital Association, Lenexa, KS) database [8]. The PHIS is a comparative pediatric database containing clinical and resource utilization data for inpatient, ambulatory surgery, emergency department, and observation unit patient encounters for 49 not-for-profit children's hospitals in the United States. Participating hospitals provide discharge and encounter data, including demographics, International Classification of Diseases codes, 9^th^ Revision and 10^th^ Revision (ICD-9 and ICD-10), Current Procedural Terminology (Codes) CPT, LOS, and discharge data. Data are deidentified at the time of submission and are subjected to bimonthly reviews and quarterly data quality reports, through a joint effort between the Child Health Corporation of America and participating hospitals [9]. The ICD codes, CPT codes, and PHIS definitions used for this study are located in Supplemental Table 1.

The University of Arkansas for Medical Sciences Institutional Review Board considered this study exempt as the data was deidentified at the time of data submission and review.

### 2.2. Patient Population

The PHIS database was queried for patients aged 3 weeks to 1 year between January 2008 and October 2020, with a diagnosis of IH requiring inpatient admission. Inclusion and exclusion diagnoses were defined based on ICD-9 and ICD-10 codes. Excluded diagnoses included lymphangioma, benign neoplasm of connective tissue, melanocytic nevi, benign neoplasm of other and unspecified endocrine glands, congenital nonneoplastic nevus, and other congenital malformations of peripheral vascular system. Additionally, patients who underwent direct laryngoscopy were presumed to have a subglottic or airway hemangioma and were excluded from the study population since these patients require more extensive ICU care focused less on propranolol treatment and more on acute airway management.

Patients less than 3 weeks of age were excluded to limit the number of patients with prolonged NICU admissions secondary to conditions unrelated to their IH. To limit inclusion of critically ill patients, those with a “Renal Flag”, “Urologic Flag”, “Mechanical Ventilation”, or “Infection Flag” were excluded. Additionally, patients with a “Cardiovascular Flag” were excluded due to possible concurrent management with propranolol.

### 2.3. Statistical Analysis

Data was summarized using counts and percentages for categorical variables, and median and interquartile range for continuous variables. Bivariate analysis was conducted using chi-square test and Fisher's exact for cross tabulation, while nonparametric continuous distributions were compared using Wilcoxon Rank Sum Test.

Dichotomous outcomes, such as mortality and readmission, were analyzed in unadjusted and adjusted models using generalized estimating equations (GEE) type of model for clustered on hospital to account for correlated data. Length of stay was modeled with a generalized linear model for gamma distributed data, log link, and repeated measures on subjects. Odds ratios and 95% confidence intervals were calculated for all predictors.

Admission trends for IH were modeled using a generalized linear model for Poisson distribution, log link, and log-transformed hospitalization as offset. Three modes were used: one with year alone (significant at *p* < .001), one with region alone (significant at *p* < .001), and one with both to assess their interaction (significant at *p* < .001).

Statistical analyses were performed in SAS Enterprise Guide v.7.1, and results were evaluated at 0.05. Visual representations were produced in GraphPad Prism v.9.

## 3. Results

### 3.1. Population

Between 2008 and 2020, a total of 3982 admission encounters for infantile hemangiomas were identified within the PHIS database. After excluding patients with prolonged NICU stay, renal and urologic abnormalities, cardiac abnormalities, need for mechanical ventilation, subglottic or airway hemangiomas, and concomitant infections, 2290 patients were identified. The majority were female (70.3%) and between 6 and 12 months of age (54.8%). The mean age was 2.91 months. Further demographic breakdown can be seen in Table 1.

### 3.2. Admission Patterns

**Figure 1:**
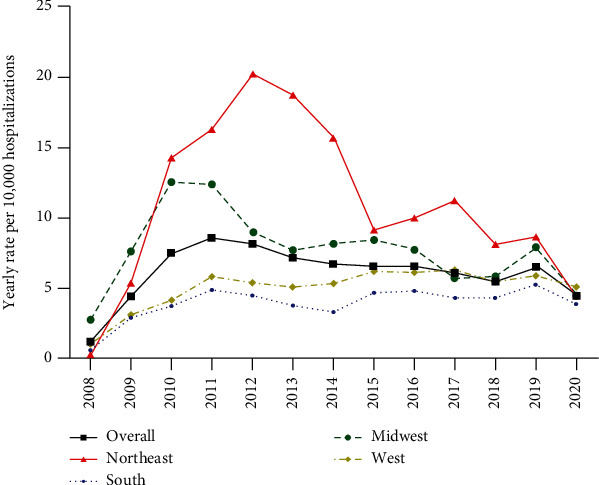
Annual rate of admission for infantile hemangiomas per 10,000 hospitalizations by geographic region. Trend of annual rate of inpatient admission per 10,000 hospitalizations for the treatment of infantile hemangiomas by geographic region from 2008 to 2020.

**Figure 2:**
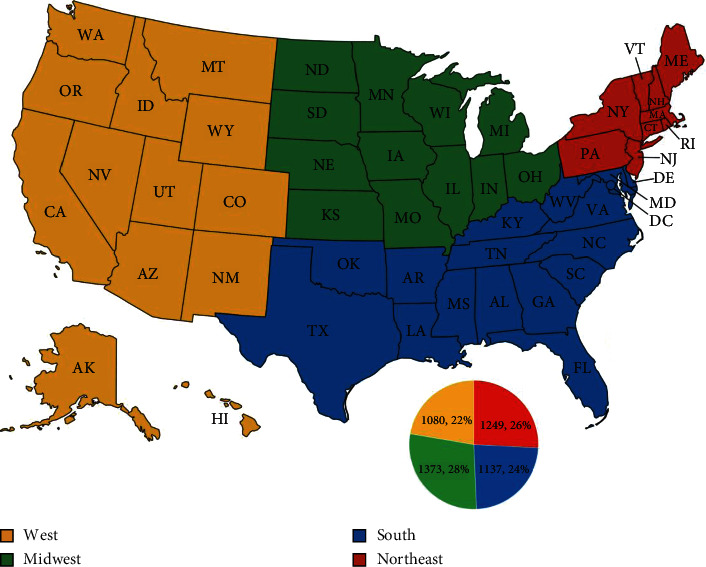
Map of the United States showing categorization of each state into geographic regions and pie chart demonstrating total IH admissions per geographic region.

Total patient admissions on an annual basis were tabulated from 2008 to 2020. The lowest value was in 2008 with 60 total admissions and the highest was 485 in 2011 (Figure 1). Overall, admissions peaked around 2011-2012 with a plateauing from 2013-18, a spike in 2019, and a drop in 2020 (Figure 1). As shown in Figure 2, admissions were grossly broken down into Northeast (Maine, Pennsylvania, etc.), South (Maryland, Florida, Texas, etc.), Midwest (North Dakota, Missouri, Ohio, etc.), and West (Montana, New Mexico, California, etc.). In the Northeast, admissions continued to rise until 2012-13 with a gradual decline. In the South, admissions gradually rose with its highest level in 2019. In the Midwest, admissions peaked in 2010-11 and declined with a small increase in 2019. In the West, admissions rose with a seemingly gradual plateau starting around 2015-16. Overall, there was an increase in rate of admissions from 2008-2011, followed by a gradual decrease. The Northeast and Midwest had higher rates of admission during the earlier years of propranolol therapy.

### 3.3. Complications Related to Treatment

Hospital stay characteristics and propranolol treatment related complications are summarized in Tables 1 and 2, respectively. No patients died and 368 (16%) required readmission after treatment. There was a total of 60 reported propranolol treatment related complications among the entire cohort.

### 3.4. Length of Stay

In univariate analysis, older age was associated with decreased length of stay (Table 3). Race, ICU admission, neurological comorbidities, airway disease, bronchospasm, beta blocker toxicity, and hyperkalemia were associated with increased length of stay. On multivariable analysis (Table 3), all factors except beta blocker toxicity remained significant. Concomitant respiratory disease doubled the risk of increased length of stay (OR 2.04 [95% CI: 1.42-2.93]). Hyperkalemia was associated with a 1.86 fold increase (95% CI: 1.08-3.20) risk of prolonged LOS.

### 3.5. Readmission

Admit age, race, female gender, neurologic comorbidities, and respiratory diseases were associated with readmission rate on univariate analysis (Table 4). In multivariate analysis, neurologic conditions (OR 2.87 [95% CI: 1.76-4.67]) and respiratory comorbidities (OR 2.48 [95% CI: 1.43-4.30]) were associated with increased readmission rates (Table 4).

### 3.6. Total Cost of Admission

In univariate analysis, older age was associated with decreased total cost (Table 5). Race, ICU admission, neurologic comorbidities, respiratory comorbidities, bronchospasm, and hyperkalemia were associated with increased total cost on univariate analysis. All variables remained significant on multivariate analysis. In addition, altered mental status was found to be associated with increased total cost on multivariate analysis (Table 5).

## 4. Discussion

In this retrospective study examining 2290 patients undergoing inpatient treatment with propranolol for IHs, we found that there have been variations in admission patterns by geographical region. Overall, very few patients suffer from treatment-related side effects from propranolol therapy, potentially resulting in unnecessary admission and increased cost of care. Patients with neurological or neuromuscular conditions and those with respiratory conditions appear to be at greatest risk of both prolonged hospitalization and readmission.

Initial protocols for the use of propranolol to treat IHs suggested that patients undergo extensive pretreatment testing and hospitalization to observe and manage potential side effects. In 2011, a consensus, multidisciplinary group first recommended inpatient treatment for infants ≤8 weeks of gestationally corrected age, or any age infant with inadequate social support, or any age infant with comorbid conditions affecting the cardiovascular system, the respiratory system, including symptomatic airway hemangiomas, or blood glucose maintenance [10]. After these guidelines were instated, there was an increase in the number of hospital admissions, length of stay (LOS), and hospital charges in the immediate years following the utilization of propranolol [11]. In 2019, a task force of experts in the fields of dermatology, cardiology, hematology-oncology, otolaryngology, plastic surgery, and radiology reconvened to update treatment guidelines on IHs. This update notes that Food and Drug Administration (FDA) labeling sanctions outpatient initiation in patients >5 weeks corrected gestational age [9].

When focusing on IH admissions alone, geographic trends reveal that the Northeast and Midwestern portions of the country had higher rates of admission during the early years (2008-2013) of propranolol therapy compared to the rest of the country. This elevated rate of hospital admission in the Northeast and Midwestern regions has trended downward. Both the South and West regions had a steady rise in admissions between 2008 and 2011 before plateauing. We could speculate that change in admission guidelines regarding IH management was released, some of the geographic regions were quicker to adapt and implement new protocols, which consequently led to lower hospital admission rates for these patients. Of note, all geographic regions experienced a drop in IH admissions in 2020, which could be attributed to decreased nonemergent admissions during the COVID-19 pandemic.

While guidelines have created frameworks for inpatient admission for IH, outpatient treatment of IH with propranolol is commonplace. Randomized control trials comparing propranolol to steroid for outpatient management have shown propranolol was not inferior to steroid with respect to therapeutic effects in IH [12]. Similar efficacy has been shown in prospective studies [13]. Surveys of otolaryngologists amongst the Vascular Anomalies Task Force show frequent use of propranolol in outpatient settings [14]. For patients above 48 weeks postconceptual age, with adequate social support, and without relevant comorbid cardiac, pulmonary, or blood glucose conditions, outpatient treatment of IH can be used over inpatient admission, especially since in-office monitoring has not been shown to change outcome [15, 16].

There were no noted deaths among the study cohort, and it is well established that propranolol treatment is relatively safe [1–7, 10, 11, 17]; however, drug side effects and predisposing factors can lead to increased length of stay and readmission. We found that those who were older than 4 months in our cohort had lower risk of increased LOS, suggesting that propranolol therapy may be better tolerated by older infants. As would be expected, patients admitted to the ICU had a significant increase in LOS, which is most likely due to severity of illness, increased care, and transition from ICU to hospital floor care. An underlying neurologic comorbidity, respiratory comorbidity, or hyperkalemia during admission were all associated with an increased LOS as well. This is likely due to the fact that propranolol has both central nervous system and respiratory side effects in which these patients with underlying conditions may be more predisposed to experiencing, resulting in longer hospitilization [6, 7, 10]. Readmission rate for our cohort of IH patients being treated with propranolol was approximately 16%. For comparison, the all cause pediatric hospital readmission in the United States in 2016 was 7.02% [13]. Again, both neurologic and respiratory comorbidities were found to be significant predictors of readmission.

While cost was similar to other pediatric admissions [14], the average cost of admission was $5185 and the total cost for all IH admissions was $11.8 million. With so few patients experiencing propranolol-related complications, there is potential in reducing the rate of these admissions to decrease overall cost of treatment for IHs. Outpatient cost for IH treatment with propranolol was $138, which increased to $828 after the addition of an echocardiogram compared to $2603 for a single hospital day and increased to $2843 with the addition of an echocardiogram [18]. It is clear that there is a strong case for outpatient management for IH treated with propranolol, when appropriate. In addition to established guidelines for admitting patients younger than 8 weeks, underlying comorbid conditions should be considered in determining whether outpatient therapy would be appropriate. As this study found, those patients more at risk for increased LOS and readmission were those with respiratory and neurologic comorbidities and may do better with inpatient therapy.

## 5. Conclusion

Overall, admission and admission rates have trended downward from their peak in 2010-2011 with the Northeast and Midwest geographic regions have seen significant decreases in rate of admissions for IH. Very few patients experience treatment related side effects from propranolol therapy. Neurologic and respiratory comorbidities are associated with increased length of stay and readmission.

## Figures and Tables

**Table 1:** Demographics and hospital stay characteristics for infantile hemangioma admissions.

	Number of patients (%)
Total	2290
Female	1610 (70.3)
Age	
3 weeks to 2 months	191 (8.3)
2 months to 4 months	232 (10.1)
4 months to 6 months	613 (26.8)
6 months to 1 year	1254 (54.8)
Race	
White	1444 (63.1)
Black	97 (4.2)
Asian	68 (3.0)
Hispanic or Latino	392 (17.1)
Other	289 (12.6)
Cost in US dollars per admission	$5,158.4 [$3,258.5-$8,560.1]
Neurologic and neuromuscular flag	90 (3.9)
Respiratory flag	69 (3.0)
Hospital stay	
Mortality	0 (0.0)
Average LOS (days) [CI]	2 [2, 3]
ICU admission	213 (9.30)
Readmission	368 (16.07)

Abbreviations: length of stay = LOS; intensive care unit = ICU.

**Table 2:** Propranolol treatment related complications.

	Number of patients (%)
Hypoglycemia	11 (0.48)
Bronchospasm	1 (0.04)
Hypotension	20 (0.87)
Altered mental status	10 (0.44)
Asthma exacerbation	0 (0.00)
Beta blocker toxicity	11 (0.48)
Hyperkalemia	7 (0.31)

**Table 3:** Odds ratios for length of stay of inpatient infantile hemangioma patients.

	Unadjusted OR (95% CI)	Adjusted OR (95% CI)
Female	1.00 (0.88-1.12)	0.99 (0.89-1.10)
Age		
3 weeks to 2 months	[reference]	[reference]
2 months to 4 months	0.91 (0.78-1.07)	0.94 (0.81-1.10)
4 months to 6 months	0.82 (0.69-0.97)^∗^	0.83 (0.71-0.97)^∗^
6 months to 1 year	0.71 (0.62-0.81)^∗^	0.70 (0.62-0.78)^∗^
Race		
White	[reference]	[reference]
Black	1.25 (1.06-1.48)^∗^	1.20 (1.02-1.41)^∗^
Asian	1.01 (0.77-1.33)	0.99 (0.72-1.34)
Hispanic or Latino	1.14 (1.00-1.23)	1.09 (0.97-1.24)
Other	1.25 (0.95-1.63)	1.23 (0.93-1.64)
ICU admission	1.43 (1.17-1.75)^∗^	1.31 (1.09-1.59)^∗^
Neurologic and neuromuscular flag	1.27 (1.07-1.52)^∗^	1.34 (1.12-1.62)^∗^
Respiratory flag	2.09 (1.44-3.03)^∗^	2.04 (1.42-2.93)^∗^
Hypoglycemia	1.83 (0.96-3.48)	1.21 (0.78-1.88)
Bronchospasm	1.38 (1.30-1.47)^∗^	1.37 (1.22-1.55)^∗^
Hypotension	1.00 (0.81-1.24)	1.00 (0.82-1.22)
Altered mental status	1.10 (0.83-1.48)	0.97 (0.72-1.31)
Beta blocker toxicity	1.29 (1.00-1.66)^∗^	1.26 (0.97-1.63)
Hyperkalemia	2.18 (1.47-3.23)^∗^	1.86 (1.08-3.20)^∗^

^∗^ = *p* < 0.05. Abbreviation: intensive care unit = ICU.

**Table 4:** Odds ratios for readmissions of inpatient infantile hemangioma patients.

	Unadjusted OR (95% CI)	Adjusted OR (95% CI)
Female	0.80 (0.65-0.98)^∗^	0.82 (0.64-1.05)
Age		
3 weeks to 2 months	1 [reference]	1 [reference]
2 months to 4 months	1.07 (0.85-1.34)	1.03 (0.78-1.36)
4 months to 6 months	1.38 (1.03-1.84)^∗^	1.35 (0.93-1.96)
6 months to 1 year	1.19 (0.86-1.64)	1.06 (0.69-1.61)
Race		
White	1 [reference]	—
Black	1.42 (0.92-2.19)	—
Asian	1.06 (0.61-1.84)	—
Hispanic or Latino	1.36 (1.06-1.74)^∗^	—
Other	0.79 (0.56-1.13)	—
ICU admission	1.09 (0.79-1.50)	0.97 (0.65-1.45)
Neurologic and neuromuscular flag	2.09 (1.49-2.93)^∗^	2.87 (1.76-4.67)^∗^
Respiratory flag	1.85 (1.23-2.78)^∗^	2.48 (1.43-4.30)^∗^
Hypoglycemia	0.56 (0.09-3.73)	0.39 (0.05-3.22)
Bronchospasm	—	—
Hypotension	1.56 (0.73-3.36)	—
Altered mental status	1.87 (0.72-4.86)	1.49 (0.35-6.27)
Beta blocker toxicity	0.56 (0.09-3.67)	0.64 (0.08-521)
Hyperkalemia	0.89 (0.14-5.47)	0.46 (0.05-4.08)

^∗^ = *p* < 0.05. Abbreviation: intensive care unit = ICU.

**Table 5:** Odds ratios for total costs of inpatient infantile hemangioma patients.

	Unadjusted OR (95% CI)	Adjusted OR (95% CI)
Female	1.03 (0.91-1.16)	1.04 (.091-1.18)
Age		
3 weeks to 2 months	1 [reference]	1 [reference]
2 months to 4 months	0.87 (0.74-1.02)	0.88 (0.74-1.04)
4 months to 6 months	0.82 (0.69-0.97)^∗^	0.91 (0.74-1.11)
6 months to 1 year	0.76 (0.63-0.91)^∗^	0.87 (0.71-1.06)
Race		
White	1 [reference]	1 [reference]
Black	1.21 (1.03-1.43)^∗^	1.30 (1.07-1.57)^∗^
Asian	1.05 (0.74-1.50)	1.09 (0.81-1.46)
Hispanic or Latino	1.26 (1.09-1.45)^∗^	1.27 (1.09-1.47)^∗^
Other	1.35 (1.01-1.80)^∗^	1.29 (1.00-1.67)^∗^
ICU admission	1.75 (1.42-2.14)^∗^	1.93 (1.60-2.31)^∗^
Neurologic and neuromuscular flag	2.23 (1.87-2.66)^∗^	2.19 (1.86-2.59)^∗^
Respiratory flag	2.47 (1.66-3.68)^∗^	2.42 (1.65-3.57)^∗^
Hypoglycemia	1.01 (0.60-1.73)	1.78 (0.74-4.29)
Bronchospasm	2.00 (1.75-2.29)^∗^	2.11 (1.98-2.25)^∗^
Hypotension	0.88 (0.66-1.18)	0.89 (0.65-1.24)
Altered mental status	1.16 (0.86-1.55)	1.69 (1.11-2.58)^∗^
Beta blocker toxicity	1.26 (0.95-1.67)	1.24 (0.93-1.64)
Hyperkalemia	2.12 (1.23-3.66)^∗^	3.11 (1.94-4.99)^∗^

^∗^ = *p* < 0.05. Abbreviation: intensive care unit = ICU.

## Data Availability

Data is available upon request. Please contact gtrichter@uams.edu if more information is required.

## Abbreviations

LOS: Length of stay
IH: Infantile hemangioma
PHIS: Pediatric Health Information System®.

## Supplementary Materials

Supplementary Materials Supplementary Table 1. ICD codes, CPT codes, and PHIS data dictionary.
