# Supplementary material for: Infantile Hemangioma Treated with Propranolol Readmission Trends, Complications of Therapy, and Cost: A PHIS Database Study
Source: Int J Pediatr. 2022 Sep 9;2022:4423558. doi: 10.1155/2022/4423558 (PMC9481381; doi:10.1155/2022/4423558)
Supplement: Supplementary Materials — Supplementary Table 1. ICD codes, CPT codes, and PHIS data dictionary. [file 4423558.f1.docx]

**Supplementary Table 1. ICD Codes, CPT Codes, and PHIS Data Dictionary**

| **Object Name** | **Description** |
| --- | --- |
| **Infantile Hemangioma** | **ICD 9:** 228.00-.04; 228-.09  **ICD 10:** D18.00, D18.01,D18.02, D18.03, D18.09 |
| **Lymphangioma** | **ICD-9:** 228.1  **ICD-10:** D18.1 |
| **Benign Neoplasm of Connective Tissue** | **ICD-9:** 215.0-.8  **ICD-10:** D210-D2120 |
| **Melanocytic Nevi** | **ICD-9:** 216.0-.9  **ICD-10:** D220-D2272 |
| **Benign Neoplasm of Other and Unspecified Endocrine Glands** | **ICD-9:** 227.0-.9  **ICD-10:** D35.0-D35.9 |
| **Congential Non-Neoplastic Nevus** | **ICD-9:** 757.32  **ICD-10:** Q82.5 |
| **Other Congenital Malformations of Peripheral Vascular System** | **ICD-9:** 757.32  **ICD-10:** Q82.5 |
| **Direct Laryngoscopy** | **CPT:** 31571 |
| **Renal and Urologic Flag** | Object is set to Y if the patient has an ICD Code in the following subcategories: Congenital anomalies, Chronic renal failure, Other, Chronic bladder diseases, Devices or Transplantation. (Based on Chris Feudtner, James A Feinstein, Wenjun Zhong, Matt Hall and Dingwei Dai, 'Pediatric complex chronic conditions classification system version 2: updated for ICD-10 and complex medical technology dependence and transplantation', BMC Pediatrics 2014, 14:199) Otherwise the object is set to N. |
| **Mechanical Vent Flag** | Identifies if a patient received a mechanical vent during this episode of care. Y=Patient had an ICD-9 Px code of 96.70, 96.71 or 96.72, ICD-10 Px code of 5A1935Z, 5A1945Z or 5A1955Z or a charge mapped to CTC code 521166 or 521169. N=No mechanical vent. (See PHIS website) |
| **Infection Flag** | Identifies an infection as defined by the ICD codes recorded that trigger this flag. Y=Patient had a diagnosis that was considered an infection. N=No infection. (See PHIS website) |
| **Cardiovascular Flag** | Object is set to Y if the patient has an ICD Code in the following subcategories: Heart and great vessel malformations, Endocardium Diseases, Cardiomyopathies, Conduction disorders and dysrhythmias, Dysrhythmias, Devices or Transplantation. (Based on Chris Feudtner, James A Feinstein, Wenjun Zhong, Matt Hall and Dingwei Dai, 'Pediatric complex chronic conditions classification system version 2: updated for ICD-10 and complex medical technology dependence and transplantation', BMC Pediatrics 2014, 14:199) Otherwise the object is set to N. |
| **Neurologic and Neuromuscular Flag** | Object is set to Y if the patient has an ICD Code in the following subcategories: Brain and spinal cord malformations, Mental retardation, Central nervous system degeneration and disease, Infantile cerebral palsy, Epilepsy, Other disorders of CNS, Occlusion of cerebral arteries, Muscular dystrophies and myopathies, Movement diseases or Devices. (Based on Chris Feudtner, James A Feinstein, Wenjun Zhong, Matt Hall and Dingwei Dai, 'Pediatric complex chronic conditions classification system version 2: updated for ICD-10 and complex medical technology dependence and transplantation', BMC Pediatrics 2014, 14:199) Otherwise the object is set to N. |
| **Respiratory Flag** | Object is set to Y if the patient has an ICD Code in the following subcategories: Respiratory malformations, Chronic respiratory disease, Cystic fibrosis, Other or Transplantation. (Based on Chris Feudtner, James A Feinstein, Wenjun Zhong, Matt Hall and Dingwei Dai, 'Pediatric complex chronic conditions classification system version 2: updated for ICD-10 and complex medical technology dependence and transplantation', BMC Pediatrics 2014, 14:199) Otherwise the object is set to N. |
